# Supplementary figures and images for: Effect of Mineral Carriers on Biofilm Formation and Nitrogen Removal Activity by an Indigenous Anammox Community from Cold Groundwater Ecosystem Alone and Bioaugmented with Biomass from a “Warm” Anammox Reactor
Source: Biology (Basel). 2022 Sep 29;11(10):1421. doi: 10.3390/biology11101421 (PMC9598201; doi:10.3390/biology11101421)

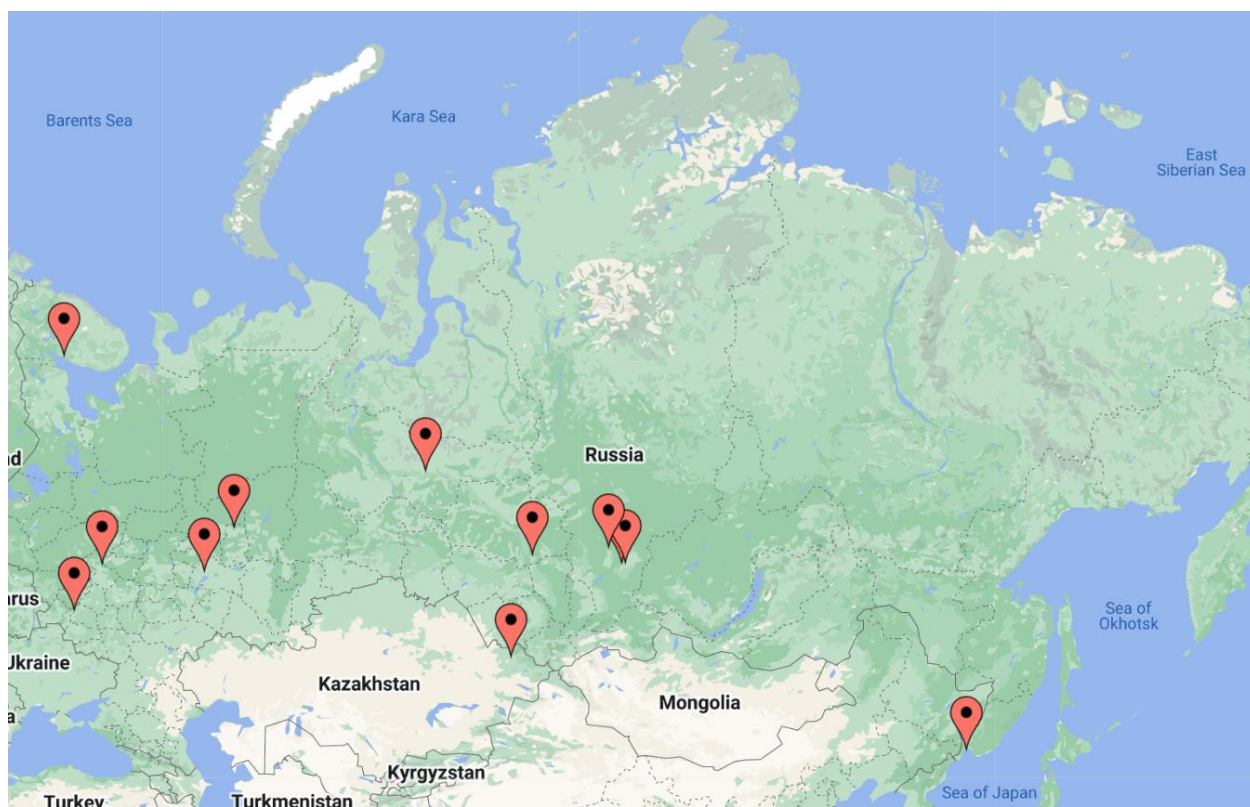

Figure S1. Location of sampling sites of the mineral carriers used in the current study.

Supplement: Supplementary file 1 [file biology-11-01421-s001.zip › biology-1925200-supplementary.pdf]
